# Delivery of Thyronamines (TAMs) to the Brain: A Preliminary Study

**DOI:** 10.3390/molecules26061616

**Published:** 2021-03-14

**Authors:** Nicoletta di Leo, Stefania Moscato, Marco Borso’, Simona Sestito, Beatrice Polini, Lavinia Bandini, Agostina Grillone, Matteo Battaglini, Alessandro Saba, Letizia Mattii, Gianni Ciofani, Grazia Chiellini

**Affiliations:** 1 Smart Bio-Interfaces, Istituto Italiano di Tecnologia, Viale Rinaldo Piaggio 34, 56025 Pontedera, Italy or nicoletta.dileo@santannapisa.it (N.d.L.); stefania.moscato@unipi.it (S.M.); agostina.grillone@gmail.com (A.G.); matteo.battaglini@iit.it (M.B.); gianni.ciofani@iit.it (G.C.); 2 The Biorobotics Institute, Scuola Superiore Sant’Anna, Viale Rinaldo Piaggio 34, 56025 Pontedera, Italy; 3 Department of Clinical & Experimental Medicine, University of Pisa, Via Savi 10, 56126 Pisa, Italy; letizia.mattii@unipi.it; 4 Laboratory of Biochemistry, Department of Pathology, University of Pisa, 56100 Pisa, Italy; marco.borso@student.unisi.it (M.B.); or ssestito@uniss.it (S.S.); beatrice.polini@farm.unipi.it (B.P.); lavinia.bandini@student.unisi.it (L.B.) alessandro.saba@unipi.it (A.S.); 5 Department of Chemistry and Pharmacy, University of Sassari, 07100 Sassari, Italy

**Keywords:** 3-iodothyronamine (T1AM), multi-target directed ligand, neurodegeneration, blood–brain barrier, high-performance liquid chromatography coupled to mass spectrometry

## Abstract

Recent reports highlighted the significant neuroprotective effects of thyronamines (TAMs), a class of endogenous thyroid hormone derivatives. In particular, 3-iodothyronamine (T1AM) has been shown to play a pleiotropic role in neurodegeneration by modulating energy metabolism and neurological functions in mice. However, the pharmacological response to T1AM might be influenced by tissue metabolism, which is known to convert T1AM into its catabolite 3-iodothyroacetic acid (TA1). Currently, several research groups are investigating the pharmacological effects of T1AM systemic administration in the search of novel therapeutic approaches for the treatment of interlinked pathologies, such as metabolic and neurodegenerative diseases (NDDs). A critical aspect in the development of new drugs for NDDs is to know their distribution in the brain, which is fundamentally related to their ability to cross the blood–brain barrier (BBB). To this end, in the present study we used the immortalized mouse brain endothelial cell line bEnd.3 to develop an in vitro model of BBB and evaluate T1AM and TA1 permeability. Both drugs, administered at 1 µM dose, were assayed by high-performance liquid chromatography coupled to mass spectrometry. Our results indicate that T1AM is able to efficiently cross the BBB, whereas TA1 is almost completely devoid of this property.

## 1. Introduction

Thyronamines (TAMs), speculated to derive from thyroid hormones (TH) through deiodination and decarboxilation, represent a novel class of signaling molecules detected in several mammal tissues, including brain, liver, pancreas and human plasma [1,2]. Among them, 3-iodothyronamine (T1AM), discovered in 2004 as the most potent agonist of trace-amine associated receptor type 1 (TAAR1) [2], has extensively been studied for almost two decades, revealing to be endowed with a wide range of bio-pharmacological properties, including metabolic reprogramming and neuroprotection [3,4,5,6,7,8,9,10,11,12].

**Scheme 1:**
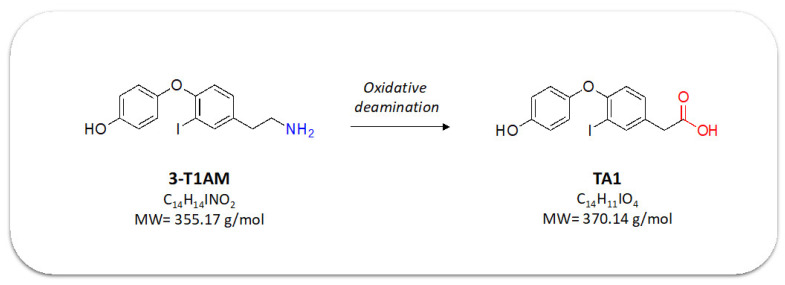
Chemical structures of 3-iodothyronamine (T1AM) and its catabolite 3-iodothyroacetic acid (TA1).

In particular, pharmacological administration of T1AM has been shown to affect reversibly and dose-dependently body temperature, cardiac functions and metabolism in rodents [13]. Moreover, intracerebral injections of T1AM at submicromolar doses (μg/Kg) appeared to increase learning ability, to reverse amnesia, to reduce pain threshold and to modulate sleep and feeding in mice [14]. However, the pharmacological effects of T1AM appeared not linearly related to the dosage and depended on the animal species and route of administration. Several degradation pathways, including oxidative deamination, deiodination, sulfation and acetylation, have been proposed to play a role in the response to T1AM. In particular, 3-iodothyroacetic acid (TA1), the product derived from T1AM oxidative deamination (Scheme 1), has been shown to significantly contribute to many of the neurological effects observed after administering T1AM to mice, including memory enhancement and reduction of pain threshold [6,15,16]. Notably, Musilli et al. [17] have shown that injections of TA1 in mice reproduces the same pro-learning effects observed after administering T1AM. Recently, TA1 anticonvulsivant and neuroprotective effects, both in vitro and in vivo, have also been shown [18], thus extending the potential of TH metabolites in the treatment of neurodegenative disorders (NDDs). 

Over the past two decades, pharmacological studies, undertaken to investigate the effect on cardiac output of exogenously administered T1AM, revealed a significant, yet reversible, negative inotropic effect in isolated working rat heart exposed to perfusion with T1AM [19]. Therefore, the heart was the first tissue to be exploited in vivo to investigate T1AM uptake and metabolism by liquid chromatography coupled to tandem mass spectrometry (LC/MS-MS) [20]. In Vitro pharmacokinetic experiments were also carried out by using cardiomyocytes [20]. In both experimental settings, a significant uptake and rapid metabolism of its corresponding TA1 were observed. Biodistribution studies after T1AM systemic administration to mice revealed that the liver is probably the primary target of T1AM biodistribution and accumulation [21], and in the liver, T1AM plays a fundamental role to maintain glucose homeostasis and to promote lipid oxidation [7].

Pharmacokinetics studies performed in liver preparations and hepatocellular carcinoma cell line displayed a rapid uptake and metabolic transformation to TA1 [4]. 

Taken together, all the information derived from previous studies points to a role of TA1 on T1AM metabolic and neurological effects. Therefore, currently, there is an ongoing effort to assess the potential of TAMs derivatives as a novel pleiotropic treatment for metabolic and neurodegenerative diseases. 

A critical aspect in the development of new drugs for the treatment of NDDs, is the presence of the blood–brain barrier (BBB), which prevents entry into the brain of most drugs in circulation [22].

The BBB is a complex structure which serves as a physical and functional barrier, regulating passive and active transport, as well as behaving as a metabolic and immunological barrier [23]. The physical barrier is represented by the endothelial cells that are linked by tight junctions (or *zonula occludens*), proteic complexes which selectively limit permeation of ions and hydrophilic agents via paracellular pathways. In particular, zonula occludens-1 (ZO-1) is the major structural protein of tight junctions, which acts as a scaffold anchoring the macromolecular junctional complexes to the cytoplasmatic actin [24]. The physical barrier is also constituted by pericytes and by feet of astrocytes. The active transport barrier results from the expression of specific membrane transporters and vesicular mechanisms for the exchange of specific essential nutrients and waste. Efflux pumps are also present in the BBB endothelial cells membrane and their role is to bond and to excrete potentially harmful agents into the extracellular environment [25,26]. On the other hand, efflux pumps are responsible also for the multidrug resistance (MDR), a phenomenon in which drugs are extruded from target cells, and thus, they are no longer effective. 

A deeply studied efflux pump is the adenosine triphosphate binding cassette (ABC) transport protein P-glycoprotein (P-gp, or ABCB1), also named multidrug resistance protein 1 (MDR1) [27,28]. 

The metabolic barrier is represented by enzymes that metabolize toxic compounds both intracellularly and extracellularly [29]. Finally, the immunological barrier results from the specialized regulation of the recruitment and transport of leukocytes and innate immune elements by the endothelium [30]. 

A direct consequence of the existence of the BBB is the clinical difficulty in delivering therapeutic compounds to the central nervous system to treat NDDs. In this paper, we investigated the ability of TAMs drug candidates, namely T1AM and its catabolite TA1, to cross the BBB, by using a previously validated in vitro model of BBB [31]. Since the membrane-associated drug transporter P-glycoprotein (P-gp) plays an essential role in drug efflux from the brain, we also evaluated the P-gp expression on the brain endothelial cell line (bEnd.3) treated with test compounds. Our results indicate that T1AM and TA1 show profound differences in the BBB permeability. T1AM efficiently crossed the BBB, whereas TA1 showed an almost negligible entry through BBB. Notably, in bEnd.3 cells, a significant uptake of T1AM was observed, followed by oxidative deamination to produce TA1, which was subsequently released through P-gp activation. 

## 2. Results 

### 2.1. In Vitro Cytotoxicity Assay

Cytotoxic effects of T1AM and TA1 after 24 h of incubation were investigated through MTT assays, by using the bEnd.3 cell line as a BBB model system and the U87MG cell line as a component of the endothelial/glial co-culture model. 

**Figure 1:**
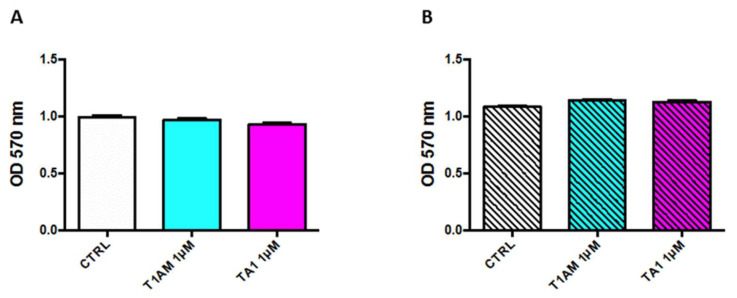
T1AM and TA1 do not promote toxicity in BBB cell models. (**A**) Mouse brain endothelial cell line (bEnd.3) viability after 24 h incubation with 1 µM T1AM, TA1 or Vehicle (DMSO 1:1000 dilution in the medium; CTRL). (**B**) Human glioblastoma cell line (U87MG) viability after 24 h incubation with 1 µM T1AM, TA1 or Vehicle (DMSO diluted 1:1000 in the medium; CTRL). Cell viability was assessed by MTT assay. For both cell preparations, values represent the mean ± SEM of three independent experiments.

As shown in Figure 1A, no significant differences between endothelial cells treated for 24 h with 1 µM T1AM or TA1 and control bEnd.3 cells were observed. The same trend was also observed for U87MG cells treated with 1 µM T1AM or TA1 for 24 h (Figure 1B).

### 2.2. Characterization of the Blood–Brain Barrier Model

**Figure 2:**
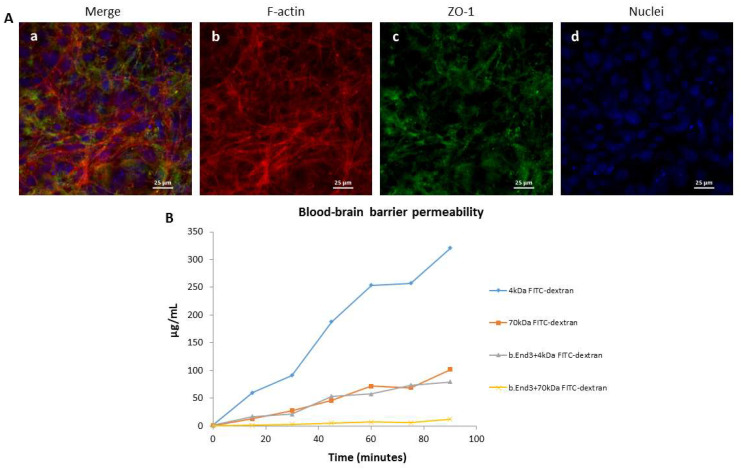
Characterization of the in vitro blood–brain barrier model. (**A**) Immunofluorescence: (**a**) merge, (**b**) F-actin in red, (**c**) ZO-1 in green, (**d**) nuclei in blue. (**B**) Permeability investigation to 4 KDa FITC-dextran and 70 KDa FITC-dextran.

**Scheme 2:**
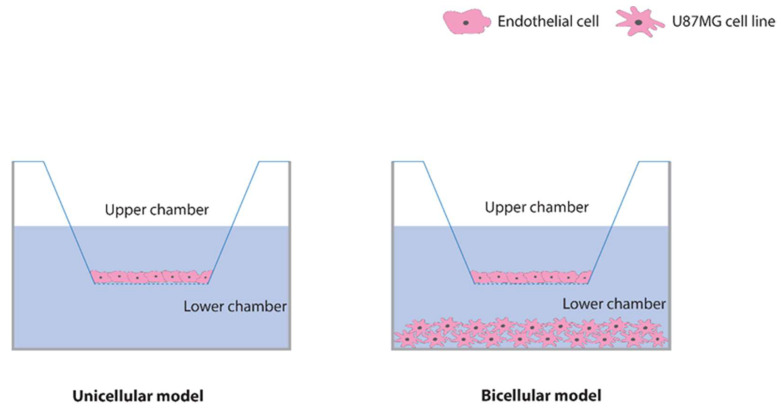
Unicellular (bEnd.3 cells) and Bicellular (bEnd.3/U87MG cells) models of the BBB.

To obtain an in vitro BBB model, bEnd.3 cells were seeded on a polyester membrane, able to sustain cell attachment and growth, which separates the upper and a lower compartment of 24-well culture plates (Scheme 2). Then, the BBB in vitro model was characterized for its biological and bioelectric properties and for its selective permeability. After 4 days from seeding, bEnd.3 cells were incubated with cAMP, hydrocortisone and 4-(3-butoxy-4-methoxybenzyl) imidazolidine-2-one to induce the tight junctions formation, which interconnect endothelial cells [31]. After the monolayer formation, an immunological approach (Figure 2A) was used to verify the presence of ZO-1 protein (in green), which is considered a marker for the BBB formation since it is crucial to assemble the tight junctions and to link them to cortical actin cytoskeleton (in red) [32,33]. 

As a further characterization of the BBB integrity, the trans-endothelial electrical resistance (TEER) was measured. In transwell cultures, values of TEER above 25 of Ω*cm^2^ are indicative of the formation of an intact barrier [34]. The TEER measurement in our blood brain barrier experimental model indicated values equal to 37.30 Ω*cm^2^. 

Finally, the paracellular selective permeability was studied by comparison with the permeability of two different molecular weights dextrans fluorescently labeled. In particular, 4 KDa and 70 kDa FITC-dextrans were used (Figure 2B). Data show that 4 KDa dextran crossing the BBB is 24.8% respect to the control (membranes pre-coated with gelatin 1% only), whereas 70 kDa dextran crossing is 11.8% respect to the control. These results confirm the higher capability of our BBB in vitro model to hinder molecules crossing, which is size-dependent. Moreover, BBB permeability was also assessed following a previously reported procedure [35]. After 1 h incubation with 2 mg/mL FITC-dextran 4 kDa or 70 kDa, we noticed that BBB permeability to 4 kDa FITC-dextran and 70 kDa FITC-dextran was 2.1 × 10^−6^ cm/s and 2.31 × 10^−7^ cm/s, respectively.

### 2.3. TAMs BBB Crossing

The ability of T1AM and TA1 to cross the in vitro model of BBB was analyzed by using a well-characterized LC-MS/MS method [11,20]. After incubating bEnd.3 cell preparations with 1 µM T1AM or TA1 Krebs-Ringer solution for 1 h at 37 °C, the media from upper and lower chambers were collected, and cell lysates were prepared according to a previously reported procedure [20]. In all experiments, cell free pre-coated membranes with gelatin 1% were also exposed to the same treatment with T1AM or TA1 and used as control. 

**Figure 3:**
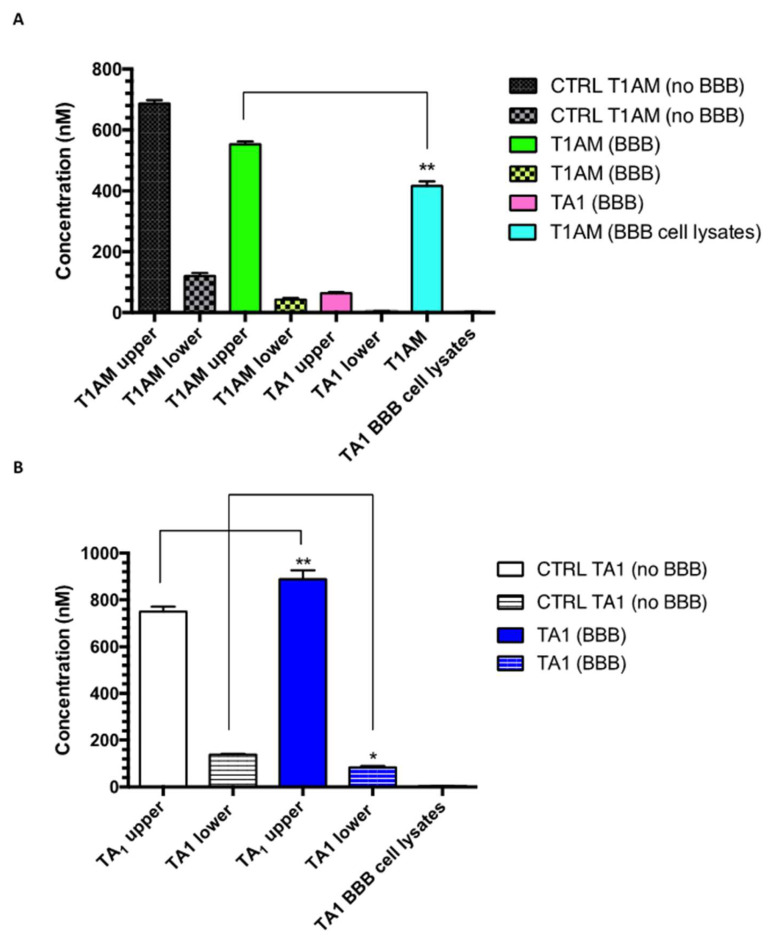
T1AM and TA1 blood–brain barrier (BBB) crossing profiles. (**A**) bEnd.3 cells seeded onto a transwell support pre-coated with 1% gelatin were incubated for 1 h with Krebs-Ringer solution containing 1 µM T1AM (upper medium). The media from upper and lower chambers were removed and analyzed by LC-MS/MS to quantified T1AM and its metabolite TA1 levels. bEnd.3 cell lysates were also analyzed to evaluate T1AM uptake and TA1 production by cellular metabolism. Cell free transwell supports exposed to the treatment with 1 µM T1AM were used as controls (CTRL). Results are expressed as mean ± SEM, *n* = 4, ** *p* < 0.005 versus T1AM (BBB) upper chamber concentration. (**B**) The same set of experiments were performed incubating bEnd.3 cells for 1 h with 1 µM TA1 in Krebs-Ringer solution (upper medium). Cell free transwell supports exposed to the treatment with 1 µM TA1 were used as controls (CTRL). Results are expressed as mean ± SEM, *n* = 4, * *p* < 0.01 versus TA1 (no BBB) upper medium concentration; ** *p* < 0.05 versus TA1 (no BBB) lower medium concentration. In all experiments, statistical significance was assigned by applying Tukey’s test.

As shown in Figure 3A, T1AM was taken up by bEnd.3 cells and catabolized to TA1, indicating that in bEnd.3 cells, amine oxidases were metabolically active. Notably, the product of T1AM catabolism, TA1, was found almost exclusively into the upper chamber, suggesting the activation of efflux pumps present in the BBB endothelial cells membrane. In bEnd.3 cell lysates, the concentration of T1AM was approximately 50% of the administered dose (** *p* < 0.005), whereas only a negligible amount of TA1 was found.

As shown in Figure 3B, after exposing bEnd.3 cells to 1 µM TA1, a very reduced uptake was observed. Indeed, after 1 h of treatment, the concentration of TA1 detected in the upper chamber was significantly higher as compared to control cells (* *p* < 0.01). The amount of TA1 found in bEnd.3 cell lysates was below the limit of detection (LOD), namely 0.3 nM [20]. 

Taken together, our results revealed a reduced TA1 cellular uptake, probably due to the absence of specific receptors on bEnd.3 cells surface, which enable cellular uptake and paracellular transport. 

**Figure 4:**
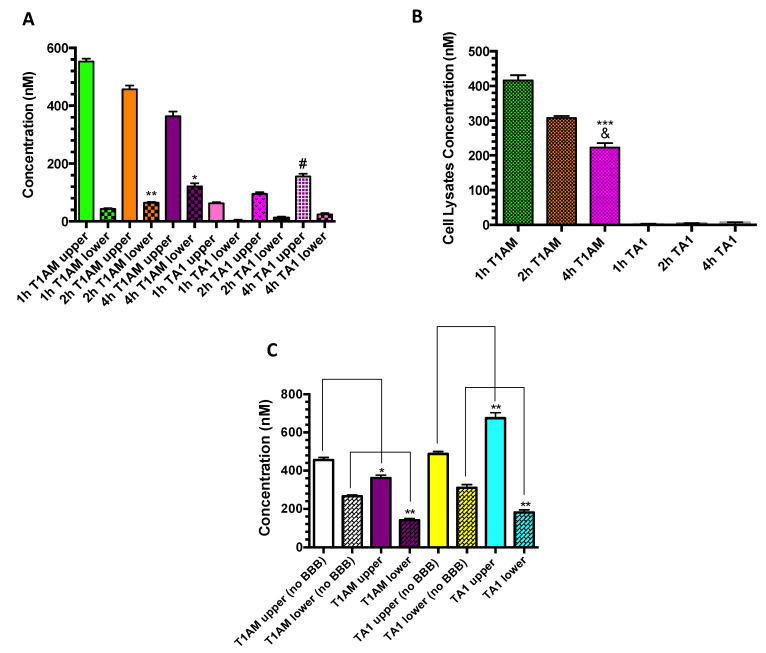
Time dependence of blood–brain barrier (BBB) permeability to T1AM and TA1. (**A**) bEnd.3 cells seeded onto a transwell support precoated with 1% gelatin were incubated for 1, 2 and 4 h with Krebs-Ringer solution containing 1 µM T1AM. The media from upper and lower chambers were removed and analyzed by LC-MS/MS to quantified T1AM BBB permeability and the efflux of intracellularly produced metabolite TA1. Results are expressed as mean ± SEM, *n* = 4, * *p* < 0.05 versus 2 h T1AM lower chamber; ** *p* < 0.01 versus 1 h T1AM lower chamber; ^#^ *p* < 0.01 versus 1 h TA1 upper chamber. (**B**) After exposure to 1, 2 and 4 h treatment with 1 µM T1AM, as described above, bEnd.3 cell lysates were prepared and analyzed by LC-M/MS to evaluate T1AM uptake and intracellular level of TA1. Results are expressed as mean ± SEM, *n* = 4, *** *p* < 0.001 versus T1AM 1 h; ^&^ *p* < 0.01 versus T1AM 2 h. (**C**) Analysis of T1AM and TA1 BBB penetrance at 4 h time point in comparison to control conditions (cell free transwell supports exposed to the treatment with 1 µM T1AM or TA1). Results are expressed as mean ± SEM, *n* = 3, * *p* < 0.05; ** *p* < 0.01. In all experiments, statistical significance was assigned by applying Tukey’s test.

To ascertain whether T1AM cellular uptake was time-dependent, the same set of experiments were repeated, increasing the incubation time of bEnd.3 cells with 1 µM test compound to 2 and 4 h. As shown in Figure 4A, a time-dependent increase of cellular permeability to T1AM was observed. Indeed, after 4 h, the detected concentration of T1AM in the medium collected from the lower chamber was approximately two-fold the concentration measured after 2 h of incubation (* *p* < 0.05), with the latter being significantly higher than that previously observed after 1 h of incubation (** *p* < 0.01). These findings are in agreement with the trend observed in T1AM cell lysates concentration (Figure 4B). After 4 h of incubation, the concentration of T1AM in bEnd.3 cell lysates was significantly reduced as compared to shorter time of incubation, namely 1 h (*** *p* < 0.001) and 2 h (^&^ *p* < 0.01) (Figure 4B). Notably, the efflux of TA1 derived from T1AM administered to bEnd.3 cell preparations followed a comparable time-dependent trend (Figure 4A). After 4 h of incubation, the concentration of TA1 detected in the medium collected from the upper chamber was approximately three times higher than that measured after 1 h of incubation (^#^ *p* < 0.01). No significant increase in TA1 concentration was observed in bEnd.3 cell lysates exposed to 1 µM T1AM for 2 and 4 h incubation time (Figure 4B). Since after 1 h treatment of bEnd.3 cells with 1 µM TA1 a very reduced uptake was previously observed, we further investigated the BBB penetrance of TA1 after 4 h incubation in comparison to T1AM BBB penetrance (Figure 4C). Our data provide evidence that increasing the incubation time to 4 h does not improve TA1 BBB penetrance property. Indeed, after 4 h of treatment, the upper chamber TA1 concentration in the presence of BBB was significantly higher as compared to control cells (TA1 upper no BBB) (** *p* < 0.01), whereas an opposite trend was observed for the BBB penetration of T1AM at the same time point. Moreover, TA1 cellular lysates concentration at the 4 h time point was below the limit of detection (data not shown).

Recent reports underlined that the induction of autophagy may at least in part contribute to the neuroprotective effects exerted by TAMs [6]. Human glioblastoma U87MG cells represent an ideal model to investigate drug induction of autophagy, because in these cells, one hallmark is mTOR upregulation [36], which in turn leads to autophagy suppression [37]. On the basis of this evidence, we generated an endothelial glial co-culture model (Scheme 2) to analyze whether T1AM and TA1 were able to reach the tumor-glial-derived U87MG cells after crossing the endothelial cell barrier, namely bEnd.3 cells. 

**Figure 5:**
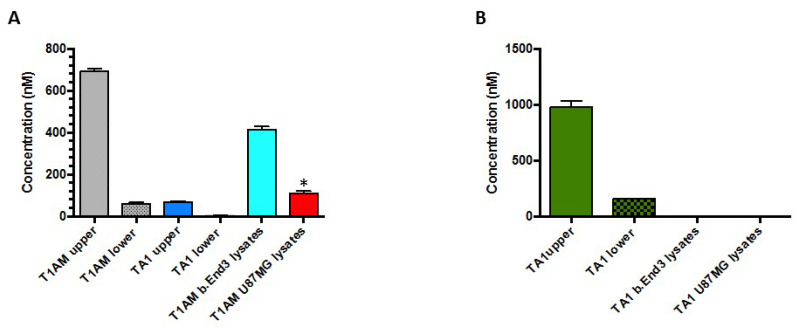
T1AM and TA1 uptake in the endothelial/tumor-glial-derived co-culture model. (**A**) bEnd.3/U87MG co-culture plates were exposed to treatment with 1 µM T1AM for 1 h. The media from the upper and lower chambers were collected and analyzed by LC-MS/MS. Cell lysates were prepared from both cell types and the levels of T1AM and TA1 were assessed. Results are expressed as mean ± SEM, *n* = 3, * *p* < 0.01 versus T1AM detected in bEnd.3 lysates. (**B**) bEnd.3/U87MG co-culture plates were exposed to treatment with 1 µM TA1 for 1 h. The media and cell lysates were analyzed by LC-MS/MS. No detectable amount of TA1 was found in U87MG cells. Results are expressed as mean ± SEM, *n* = 3.

As shown in Figure 5A, after exposure for 1 h to 1 µM T1AM, bEnd.3 cells allowed T1AM to distribute to U87MG cells, even though the concentration of T1AM detected in U87MG cell lysates was approximately four-fold lower as compared to the concentration measured in bEnd.3 cell lysates. Consistent with previous experiments carried out on bEnd.3 cells, no detection of TA1 was observed in U87MG cell lysates after exposing the bEnd.3 cell barriers to T1AM. 

When the same set of experiments were repeated by using 1 µM TA1 (Figure 5B), the analyte was completely undetectable in U87MG cell lysates, confirming its poor ability to penetrate inside cells.

### 2.4. P-Glycoprotein (P-gp) Inhibition Assay

P-glycoprotein (P-gp) is a transmembrane glycoprotein which affects the pharmacokinetics of several compounds by extruding them from cells, and ultimately, it induces multidrug resistance [38]. Since after exposing our BBB model to 1 µM T1AM, intracellularly produced TA1 was found predominantly in the upper chamber medium (Figure 3A), we hypothesized that after production, TA1 was extruded from the cells by the action of P-gp efflux pump. To prove this hypothesis, we used an immunological approach to detect the expression of P-gp in b.End.3 cells exposed to T1AM treatment. 

**Figure 6:**
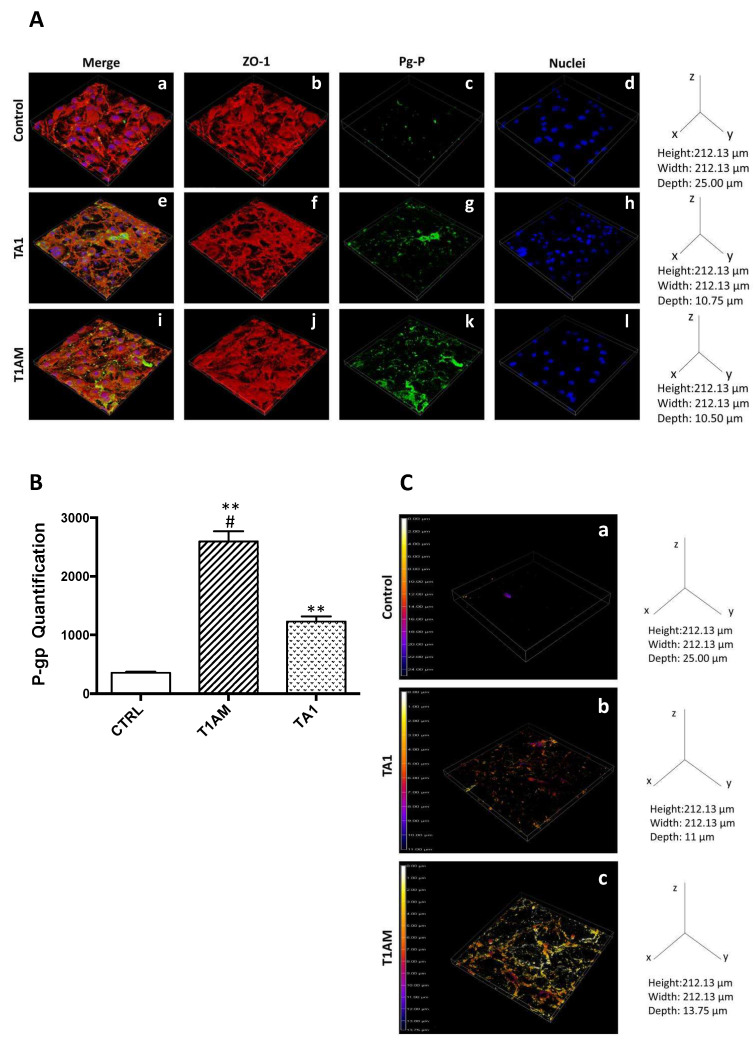
Immunofluorescence characterization of P-gp protein expression after the administration of 1 µM TA1 and T1AM. First row: Control; second row: TA1; third row: T1AM. Columns in order: merge; ZO-1 (in red); P-gp (in green); nuclei (in blue). (**A**) P-gp expression. Increased expression of P-gp was observed in bEnd.3 exposed to 1 µM TA1 (**g**) or T1AM (**k**) as compared to bEnd.3 cells treated with vehicle (DMSO 1:1000 dilution in the medium) as control (**c**). (**B**) P-gp quantitative analysis. P-gp protein expression in bEnd.3 cells measured on the maximum projections. T1AM or TA1 1 µM treatment increases P-gp expression compared to control (DMSO 1:1000 dilution in the medium). T1AM induced a significant higher increase respect to TA1. Data are reported as mean ± SEM. ** *p* < 0.01 versus CTRL, ^#^ *p* < 0.01 versus TA1. (**C**) P-gp distribution on cell surface. Color scale representation of P-gp localization from basal (blue) to apical (orange-white) cell surface. Apical membrane localization of P-gp is particularly evident on bEnd.3 cells treated with T1AM (**c**).

As shown in Figure 6A (panels a,e,i and c,g,k), treatment of bEnd.3 cells with 1 µM T1AM or TA1 induced an increased P-gp immunoreaction compared to vehicle treated bEnd.3 cells, used as control (DMSO 1:1000 dilution in the medium). Quantitative analysis confirmed a significantly higher expression of P-gp in cells treated with T1AM or TA1 respect to control, also revealing a more pronounced effect of T1AM as compared to TA1 (Figure 6B). 

Regarding the localization of Pgp, we have observed that in bEnd.3 cells, the protein is highly expressed both at the cytoplasmic level and at the cell membrane level (Figure 6A, panels a,e,i and c,g,k). As clearly displayed in Figure 6C, where the color variation from blue to white shows the P-gp expression from the basal to the apical level of the BBB, respectively, after T1AM treatment, P-gp appeared to be mainly expressed at the apical surface of the cells. 

**Figure 7:**
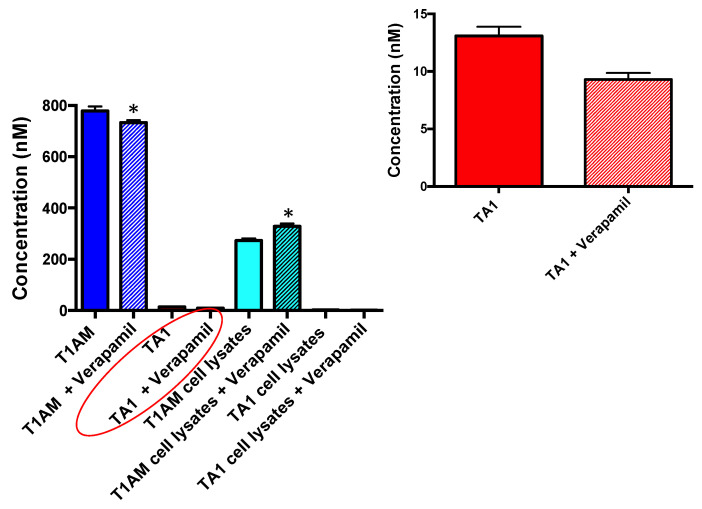
Effects of Verapamil pretreatment in bEnd.3 cells exposed to T1AM. 8 × 10^4^ bEnd.3 cells seeded in a 48-well plate were exposed to pretreatment 20 µM Verapamil followed by incubation for 1 h with 1 µM T1AM. The medium was removed and analyzed by LC-MS/MS to quantified T1AM uptake and the efflux of endogenously produced metabolite TA1 from cells. Cell lysates were also analyzed to evaluate T1AM and TA1 intracellular levels. bEnd.3 cells not exposed to Verapamil pretreatment were used as control. Results are expressed as mean ± SEM, *n* = 3, * *p* < 0.05 versus control treatment.

Once it was established that T1AM may induce increased expression of P-gp in bEnd.3 cells, we proceeded to evaluate the effect of P-gp inhibition by Verapamil [39] on T1AM uptake and metabolism in bEnd.3 cells. As shown in Figure 7, pretreatment of bEnd.3 cells with 20 µM Verapamil produced a significant increase of T1AM uptake (* *p* < 0.05) and retention (* *p* < 0.05), while decreasing the level of TA1 released from the cells and detected in the medium (* *p* < 0.05) (see the magnified panel of Figure 7). As previously observed, almost a negligible amount of TA1 was found in cell lysates both with and without pretreatment with Verapamil. Even though they are still at a preliminary level, these results seem to support our hypothesis that T1AM induces the expression of P-gp efflux pump in bEnd.3 cells, which is involved in the efflux of intracellularly produced TA1. Nevertheless, the observed increase in T1AM detected in cell lysates and decrease in TA1 released from the cell with no change in TA1 levels in cell lysates could also be explained by a reduction in the metabolism of T1AM to TA1 by Verapamil treatment. 

## 3. Discussion

Thyronamines (TAMs), a novel class of endogenous compounds assumed to derive from thyroid hormones through deiodination and decarboxylation [40], have attracted the attention of researchers for several years because of their wide spectrum of biological activities. As extensively described in recent literature [10,41] for 3-iodothyronamine (T1AM), the lead compound of this class, cryogenic, cardiac and metabolic properties have been demonstrated in several animal models. Notably, T1AM and derivatives have also shown great application potential as novel pleiotropic drugs for the treatment of dementia and NDDs [6,42]. However, one of the limiting factors in the treatment of NDDs is represented by the presence of the blood brain barrier (BBB), a complex structure that behaves like a physical barrier, protecting the brain from infections, neurotoxins and maintaining the balance of nutrients and macromolecules between the brain and the external environment, which in turn can halt the delivery of drugs to the brain [43]. 

In this paper, we examined the BBB crossing capability of T1AM and its endogenous metabolite TA1 by using murine brain endothelial cells (bEnd.3), seeded on transwell inserts as an in vitro BBB model system [44,45,46]. Initially, we tested our BBB in vitro model to ascertaining its permeability and bioelectric properties. Indeed, immunohisto-chemistry experiments and permeability assays revealed the production of the ZO-1 protein, a marker for the BBB formation, as well as a size-selective permeability, which in turn validate the functioning of our BBB model.

**Scheme 3:**
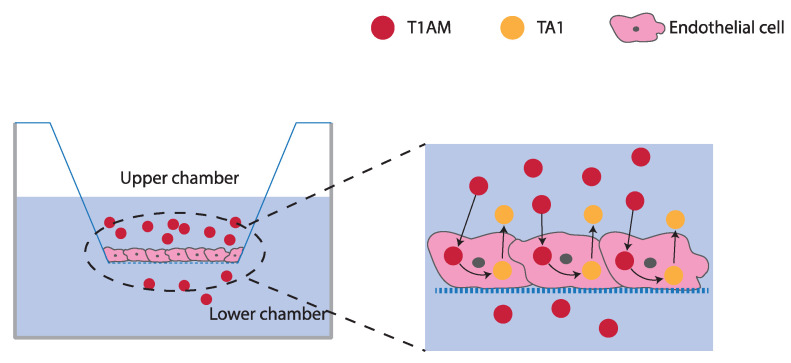
Model of T1AM import/export/metabolization in bEnd.3 cells.

Once we had validated the barrier functionality, we proceeded to test the BBB permeability to T1AM and TA1, both administered at a dosage of 1 μM. Our analysis indicated that T1AM and TA1 display significantly different BBB penetrance properties. after 1 h of incubation time, T1AM was efficiently taken up by bEnd.3 cells, since approximately half of the administered dose was found in cell lysates. As shown in Scheme 3, endothelial cells forming the BBB also rapidly metabolized T1AM to TA1, which was then released from cells, presumably through the activation of efflux pumps, and was detected in the upper chamber medium. A significant amount of T1AM was also detected in the lower chamber medium, indicating a certain ability of the compound to cross the BBB after 1 h of incubation time. 

On the contrary, administration of 1 μM TA1 did not induce any significant cellular uptake, with a measured concentration of TA1 in the upper chamber of the transwell system almost comparable to the administered dose. By examining the chemical structures of T1AM and TA1 (Scheme 1), it is possible to observe that TA1 differs from T1AM by the presence in the side chain of a carboxylic acid substituent instead of an amino group; therefore, at physiological pH, the two drugs show an opposite polarization, with the carboxylic acid substituent bearing a negative charge, which is known to be a liability for BBB permeability and CNS drug distribution. Even though this aspect may deserve additional investigation, we can also speculate the absence of specific transporters on bEnd.3 cells surface.

T1AM BBB permeability was subsequently evaluated in a time-dependent manner. To this aim, the BBB model was exposed to 1 μM of T1AM for 2 and 4 h of incubation time, and at the end of each experiment, the media collected from the upper and lower chambers, along with the relative cell lysates preparation, were analyzed by LC-MS/MS to assay the BBB permeability to T1AM, and to detect the metabolic production of TA1. Notably, a time-dependent increase of the lower chamber medium T1AM concentration was observed by LC-MS/MS analysis, indicating that a longer exposure time of bEnd.3 cells to T1AM enhances the cell permeability to T1AM. Consistently, a parallel decrease of cell lysates T1AM concentration was also observed. Notably, a time-dependent increase of the upper chamber medium concentration of endogenously produced TA1 was observed, further confirming the activation of efflux pumps after treatment of bEnd.3 cells with T1AM. 

To verify whether the permeability of our BBB model to T1AM might allow the distribution of T1AM to target glial cells, we set up an endothelial/glial co-culture transwell system by using U87MG cells. After incubating bEnd.3 cells with 1 μM T1AM for 1 h, we proceeded to detect T1AM concentration in U87MG cell lysates by LC-MS/MS analysis. Our results revealed that, after crossing the BBB, T1AM was able to reach U87MG cells, even though its concentration in U87MG cell lysates was approximately four-fold lower as compared to the concentration measured in bEnd.3 cell lysates. Consistent with previous experiments carried out on bEnd.3 cells, TA1 was not detected in U87MG cell lysates after treatment with T1AM. 

In the bEnd.3/U87MG co-culture model, the treatment with 1 µM of TA1 for 1 h resulted in the detection of extremely low levels of the analyte in both bEnd.3 and U87MG cell lysates, confirming the reduced ability of TA1 to penetrate inside the cells. 

Finally, to assess the contribution of efflux pumps on the extrusion of TA1 from endothelial cells, we focused on P-gp protein expression and inhibition by Verapamil inhibitor administration. 

The results obtained in the immunohistochemistry assays showed a significantly increased expression of P-gp in bEnd.3 cells after T1AM administration compared to TA1 or vehicle administration. Furthermore, we observed an apical surface localization of this protein mainly after treatment with T1AM.

In P-gp inhibition experiments, treatment with Verapamil caused a significant decrease in TA1 efflux from bEnd.3 (*p* < 0.05), supporting at least in part the hypothesis that P-gp might contribute to the efflux of TA1 from bEnd.3 cells, although the inhibition of T1AM metabolism by Verapamil can be equally hypothesized.

## 4. Materials and Methods

### 4.1. Cell Cultures

bEnd.3 and U87 MG cell lines were purchased by Sigma-Aldrich and were grown in DMEM (Sigma-Aldrich, St. Louis, MO, USA), supplemented with 10% FBS, 1% penicillin-streptomycin and 1% glutamine (all from Gibco) and kept at 37 °C in 5% CO_2_-humified atmosphere.

### 4.2. MTT Assay

To assess a possible TAMs cytotoxic effect, 3-(4,5 dimethylthiazole-2-yl)-2,5-diphenyl tetrazolium bromide (MTT) assay was performed on both bEnd.3 and U87MG cell lines after T1AM and TA1 administration. 

For bEnd.3 cells, 8 × 10^3^ cells were seeded in a 96-well plate and incubated at 37 °C in 5% CO_2_ humified atmosphere overnight. In order to simulate the BBB conditions (as described below), the growing medium was replaced with fresh medium containing the factors for the tight junctions induction and incubated at 37 °C and 5% CO_2_. After 24 h, the medium was removed and replaced with fresh medium containing 1 µM of T1AM or TA1, and the cells were incubated at 37 °C and 5% CO_2_ for 24 h. Then, 0.5 mg/mL MTT (Sigma-Aldrich) diluted in complete cell medium, was added. Cells were incubated at 37 °C and 5% CO_2_ for 2 h. The medium was then replaced with 100 µL of DMSO (Sigma-Aldrich) and cells were kept at room temperature for 10 min on a shaker. The absorbance was measured on a microplate photometer (HiPo, MPP−96, Biosan) at a wavelength of 570 nm. 

The effects of 1 µM T1AM or TA1 treatment for 24 h on U87MG cell viability were evaluated by using the MTT as previously described [47].

### 4.3. In Vitro BBB Model Preparation and Characterization

In all experiments, 36 × 10^3^ bEnd.3 cells were seeded onto a transwell support (400 nm pore size polyester inserts, Life Technologies) precoated with 1% gelatin (Sigma-Aldrich), in a 24-well plate. Cells were then incubated at 37 °C with 5% CO_2_ for 5 days to form an endothelial cells monolayer on the membrane. Twenty-four hours before treatment with TAMs, the cell medium from both upper and lower chamber was replaced with fresh medium [31] containing 250 μM of 8-(4 chlorophenylthio adenosine 3′,5′ cyclic monophosphate sodium salt (pCPT-cAMP) (Sigma-Aldrich), 17.5 μM of 4-(3-butoxy-4-methoxybenzyl) imidazolidine-2-one (Sigma-Aldrich) and 550 nM of hydrocortisone (Sigma-Aldrich) to promote the tight junctions formation [48]. 

Before performing treatment experiments, the integrity of the bEnd.3 cells monolayer, the bioelectric properties and the permeability to solutes were investigated. To this aim, the upper and lower chambers of the transwell inserts were filled with DMEM, serum and phenol-red free, and incubated for 30 min at 37 °C. Then, transendothelial electrical resistance (TEER) was measured by using Millicell ERS-2 (Electrical Resistance System, Millipore, Bedford, MA, USA).

For permeability studies, the diffusion of 4 kDa FITC-dextran and 70 kDa FITC-dextran (Sigma-Aldrich) across the bEnd.3 monolayer was evaluated. For the tracers, 300 μL of 2 mg/mL solutions in phenol red free were prepared. Cells were incubated at 37 °C, and 100 μL of the medium were taken up from the basolateral chambers and transferred into 96-well black plates to measure the fluorescence through a microplate reader (Victor3, excitation 485 nm, emission 544 nm; PerkinElmer, Turku, FIN), with readings every 15 min up to 90 min. The diffusion of the fluorescent molecules through the BBB model was compared to the diffusion of the same marker across control membranes pre-coated with gelatin 1%.

The BBB models, prepared as described above, were also investigated for ZO-1 formation by immunofluorescence followed by confocal imaging. After fixation with PFA 4% for 20 min at 4 °C and permeabilization with Triton X−100 0.1% for 20 min at room temperature (RT), cells were blocked with 10% goat serum solution for 45 min at RT, and incubated for 3 h RT with primary antibody ZO-1 (Invitrogen, Thermo Fisher, Rodano (MI), Italy) diluted 1:200 in 10% goat serum. After rinsing three-times with PBS, BBB models were incubated for 45 min at 37 °C with a staining solution containing Atto 488 goat anti-rabbit secondary antibody (Sigma-Aldrich) 1:200, TRITC-phalloidin (Sigma-Aldrich) 1:100, and Hoechst 33342 (Invitrogen) 1:1000 in 10% goat serum. After rinsing three times with PBS, images were acquired by confocal microscope (C2s, Nikon, Florence, Italy).

### 4.4. T1AM and TA1 BBB Crossing Experiments

After having demonstrated the in vitro formation of the BBB, the medium from the upper and lower chambers was removed. In the upper chambers, a Krebs-Ringer solution containing T1AM or TA1 at a final concentration of 1 µM was added, whereas pure Krebs-Ringer solution was added into the lower chambers and the plates were incubated for 1 h at 37 °C. The diffusion of T1AM or TA1 across empty membranes pre-coated with 1% gelatin was used as control.

The medium from the lower and upper chambers was separately collected and placed at −20 °C. The BBB cell lysates were also prepared to evaluate the cellular uptake of the compounds under investigation. To this end, the BBB cell lysates were rinsed once with Krebs and placed at −20 °C for 30 min. Then, 100 µL of NaOH 0.1 N were added to lyse BBB cells, and after scraping 10 µL of HCl 1 N were added to neutralize the lysates. Cell lysates were transferred into Eppendorf tubes (Sigma-Aldrich, St. Louis, MO, USA) and centrifuged at 600× *g* at 4 °C for 30 min. The supernatants were collected and kept at −20 °C.

To quantify the BBB permeability to T1AM or TA1, the media collected from the lower and upper chambers, respectively, were exposed to LC-MS/MS analysis by using a previously established procedure that allows the simultaneous detection of T1AM and TA1 in each sample [11,20]. T1AM and TA1 intracellular concentrations were also assayed by exposing cell lysate samples to the same LC-MS/MS method.

Briefly, aliquots (0.1 mL) from each sample collection were spiked with 10 μL of a suitable mixture of internal standards (deuterated T1AM and TA1). After adding methanol (0.4 mL), the samples were shaken for 10–15 min and centrifuged at 22,780× *g* for 10−12 min. The supernatant was dried under a gentle stream of nitrogen, reconstituted with water/methanol mixture (70/30 by volume) and injected into the LC-MS-MS system. The latter included an Agilent 1290 UHPLC system (Santa Clara, CA, USA) coupled to an AB-Sciex API 4000 triple quadrupole mass spectrometer (Concord, ON, Canada). 

### 4.5. Endothelial/tumor-Glial-Derived Co-Cultures

To evaluate the uptake by U87MG cells of TAMs after crossing the BBB, we generated an endothelial/glial co-culture. To this aim, we set up the BBB model as described in paragraph 4.3. 

Twenty-four hours before performing the BBB crossing experiments, 5 × 10^4^ U87MG cells were seeded on the bottom of a fresh 24 well plate, and incubated overnight at 37 °C. After 24 h, the culture medium was removed, and after rinsing three times with 500 μL of Krebs-Ringer solution, 1.2 mL of Krebs-Ringer solution were added to U87MG cells. Meanwhile, after rinsing with Krebs-Ringer solution, BBB cells were transferred to the 24 well plate containing U87MG cells. 1 µM T1AM or TA1 were added to the upper chamber containing BBB cells, and the plate was incubated for 1 h at 37 °C. Then, the media from the lower and upper chambers were separately collected and placed at −20 °C. Distinct cell lysates were prepared from both bEnd.3 and U87MG cells following the same procedure described above. 

### 4.6. P-gp Immunofluorescence

5 × 10^4^ bEnd.3 cells were seeded on WillCo^®^ glass bottom dishes in complete cell medium and incubated at 37 °C and 5% CO_2_ for 24 h. Then, cells were treated with 1 µM T1AM, TA1 or vehicle (DMSO 1:1000 dilution) in a Krebs-Ringer solution for 1 h, fixed with 1% formaldehyde in phosphate buffer solution 1X (PBS) for 10 min at 4 °C. For immunofluorescence experiments, cells were treated with 0.2% triton X−100/PBS for 10 min at RT. Then, after 1 h in blocking solution (BS) (PBS, 0.1% Tween, 0.25% BSA in PBS) at RT, samples were incubated overnight at 4 °C with the primary antibody (anti-P-glycoprotein P7965; Sigma-Aldrich) diluted 1:100 in BS. Samples were then washed three times in PBS, and incubated for 90 min with fluorescent anti-rabbit secondary antibody diluted 1:250 in PBS (Alexa Fluor^®^ 568, Life Technologies). From this step to the end, samples were kept in the dark. After a wash in PBS for 10 min, the samples were rinsed three times with PBS 1X for 5 min. Dapi 1 µg/mL in PBS 1X was added for 15 min and then washed three times with PBS. Images were acquired by confocal microscope (C2s, Nikon).

Quantitative image analysis was performed using ImageJ software (NIH, Bethesda, MD, USA). Briefly, the maximum projection of ten independent microscopic fields for each experimental class were performed. Then, the maximum projections were analyzed by measuring the mean value of their intensity. Data were analyzed for statistics as described below. 

### 4.7. P-gp Inhibition Assay 

8 × 10^4^ bEnd.3 cells were seeded in a 48-well plate and incubated at 37 °C and 5% CO_2_. After 24 h, the medium was removed and cells were stimulated to form the tight junction as previously described. The plate was kept overnight at 37 °C and 5% CO_2_. The medium was replaced by fresh medium added with Verapamil 20 µM (Sigma-Aldrich) to inhibit the P-gp protein activity. Cells were kept overnight at 37 °C and 5% CO_2_, aftert which the medium was removed and a Krebs-Ringer solution containing 1 µM T1AM or TA1 was added. Cells were incubated for 1 h. Then, the medium from each well was collected and the plate was placed at −20 °C for 30 min. NaOH 0.1N and HCl 1N were added to lyse the cells, as previously described. Cell lysates were transferred into Eppendorf tubes and centrifuged at 600× *g* for 30 min. The supernatants were collected and placed at −20 °C. T1AM and TA1 supernatant and cell lysates concentrations were also assayed by using the same LC-MS/MS method described above.

### 4.8. Statistical Analysis

Statistical analyses were performed using GraphPad Prism version 6.0 for Windows (GraphPad Software, San Diego, CA, USA). Data were subjected to a one-way analysis of variance for mean comparison, and significant differences among different treatments were calculated according to Tukey’s HSD (honest significant difference) multiple range test. Data are reported as mean ± SEM. Differences at *p* < 0.05 were considered statistically significant. 

### 4.9. Drugs

T1AM was purchased from Sigma-Aldrich (St. Louis, MO, USA); TA1 was kindly provided by Dr. Scanlan TS. Aliquots were stored at −20 °C in DMSO as a 200 mM stock solution and diluted to the desired concentration in Krebs-Ringer solution. The Krebs-Ringer solution was prepared as follows (in mM): 120 sodium chloride, 5 potassium chloride, 2 calcium chloride, 1 magnesium chloride, 25 sodium bicarbonate and 5,5 D-glucose.

## 5. Conclusions

Even though still at a preliminary level, the results of our study demonstrate for the first time that T1AM has the ability to cross an in vitro model of BBB in a time-dependent fashion, and to distribute to brain target cells. After uptake, murine brain endothelial cells (bEnd.3) metabolize T1AM to its main catabolite TA1, which is promptly released from the cells by the activation of the P-gp efflux pump. In bEnd.3 cells, an almost negligible uptake of exogenously administered TA1 was also observed. Taken together, these results contribute to confirm the potential of T1AM as a novel drug for the treatment of NDDs, whereas for TA1, the observed reduced BBB penetrance suggests the need to exploit a novel strategy to improve the drug delivery to the brain. 

## Data Availability

The data that support the findings of this study are available from the corresponding author upon reasonable request.

## Sample Availability

Samples of the compounds are available from the authors.
